# Stability of healthy subgingival microbiome across space and time

**DOI:** 10.1038/s41598-021-03479-2

**Published:** 2021-12-14

**Authors:** Ryan Tamashiro, Leah Strange, Kristin Schnackenberg, Janelle Santos, Hana Gadalla, Lisa Zhao, Eric C. Li, Emilie Hill, Brett Hill, Gurjit Sidhu, Mariana Kirst, Clay Walker, Gary P. Wang

**Affiliations:** 1 Division of Infectious Diseases and Global Medicine, Department of Medicine, College of Medicine, University of Florida, Gainesville, FL USA; 2 Department of Periodontology, College of Dentistry, University of Florida, Gainesville, FL USA; 3 Department of Endotontics, College of Dentistry, University of Florida, Gainesville, FL USA; 4 Department of Oral Biology, College of Dentistry, University of Florida, Gainesville, FL USA; 5 Medical Service, North Florida/South Georgia Veterans Health System, Gainesville, FL USA

**Keywords:** Microbial communities, Pathogens

## Abstract

The subgingival microbiome is one of the most stable microbial ecosystems in the human body. Alterations in the subgingival microbiome have been associated with periodontal disease, but their variations over time and between different subgingival sites in periodontally healthy individuals have not been well described. We performed extensive, longitudinal sampling of the subgingival microbiome from five periodontally healthy individuals to define baseline spatial and temporal variations. A total of 251 subgingival samples from 5 subjects were collected over 6–12 months and deep sequenced. The overall microbial diversity and composition differed significantly between individuals. Within each individual, we observed considerable differences in microbiome composition between different subgingival sites. However, for a given site, the microbiome was remarkably stable over time, and this stability was associated with increased microbial diversity but was inversely correlated with the enrichment of putative periodontal pathogens. In contrast to microbiome composition, the predicted functional metagenome was similar across space and time, suggesting that periodontal health is associated with shared gene functions encoded by different microbiome consortia that are individualized. To our knowledge, this is one of the most detailed longitudinal analysis of the healthy subgingival microbiome to date that examined the longitudinal variability of different subgingival sites within individuals. These results suggest that a single measurement of the healthy subgingival microbiome at a given site can provide long term information of the microbial composition and functional potential, but sampling of each site is necessary to define the composition and community structure at individual subgingival sites.

## Introduction

The human microbiome is composed of complex microbial communities that perform essential physiologic functions for the health of the host^1,2^. Alterations of the microbiome in different body habitats have been associated with a number of diseased states^3–9^. These alterations disrupt health-promoting functions, which may lead to or exacerbate disease^3–5,10^. While many studies have examined the differences in microbial communities between health and disease, few have focused on the biogeography and temporal variations of the healthy microbiome.

Studies in healthy volunteers have shown that within each body habitat, microbial communities encode metabolic functions that are shared among individuals^2,11–13^. However, their microbial composition varies widely and is highly individualized^2,11,12,14^. Temporal analysis of the human microbiome suggest that microbiome is relatively stable over time^11,12,14–18^, but the degree of stability varies between different body habitats^12,13,15,19^ and differs among individuals^14,15,19^. These observations suggest that temporal dynamics of the microbiome is influenced by host and environmental factors such as life stage, host genetics, and lifestyle. Longitudinal studies suggest that temporal stability of microbiome correlate with microbial diversity^13–15^, but the relationship between diversity and stability across different body habitats is less clear^13,15^. Compared to open environments, communities in closed environments may fluctuate less over time as they are exposed to fewer potential colonizers and environmental perturbations. For example, oral and gut communities have shown a higher degree of temporal stability compared to the more variable skin microbiomes^12,13,15^.

The oral cavity harbors one of the most stable microbiomes in the human body^11–13,15^. It contains several different sub-habitats including the tongue, hard palate, dental surfaces, and subgingival space, with each sub-habitat hosting a unique microbiome^2,13,19^. Due to the ease of sampling, oral rinse, saliva, and tongue swabs are frequently used to sample the oral microbiome, and thus the temporal variations of salivary, tongue, and oral wash microbiota have now been well described^11–13,15,19–21^. However, the spatial and temporal variations of subgingival microbiome have not been investigated in detail.

The subgingival microbiome varies among periodontally healthy individuals but is typically dominated by five major phyla: *Firmicutes, Actinobacteria, Bacteroidetes, Fusobacteria,* and *Proteobacteria*^7,9,22^. Alterations in the subgingival microbiome have been associated with periodontal disease in cross-sectional studies. Compared to diseased sites, healthy subgingival microbiome is generally less diverse and is enriched with gram-positive commensals^7,22^. Studies have shown that subgingival microbiome in diseased sites have less inter-individual variations and are often enriched with organisms from the red and orange Socransky complex^9,22,23^, many of which belong to the *Bacteroidetes* phylum^23,24^. While differences between healthy and diseased subgingival microbiota have been well described^7–10,22^, long term stability of the healthy subgingival microbiome have not been reported. Furthermore, since many studies have pooled subgingival samples from multiple sites for analysis, the effects of spatial variation and site-to-site variability are not known.

Here, we report spatial configuration and temporal dynamics of subgingival microbiome sampled extensively from five periodontally healthy individuals. The longitudinal analysis allowed us to define inter- and intra-individual and temporal variations of the subgingival microbiome, providing a window on site-to-site variability and long-term stability within and between individuals. In addition, we identified features of the microbiome influencing temporal stability, and compared temporal dynamics of the microbiome and the predicted functional metagenome.

## Results

### Sampling and analysis of the subgingival microbiome

We sequenced subgingival microbiomes longitudinally from five periodontally healthy individuals at three or four time points over 12 months, generating 5,602,156 reads with an average of 22,409 reads per sample (range 4148–207,525). A total of 556 phylotypes were identified from 251 subgingival samples, belonging to 123 genera in 10 different phyla. Five phyla comprised over 97% of the total sequence reads: *Firmicutes* (33.3%), *Actinobacteria* (20.3%), *Bacteroidetes* (15.8%), *Proteobacteria* (15.5%), and *Fusobacteria* (12.8%). *Spirochetes*, *TM7*, *Synergistetes*, *SR1*, and *Chloroflexi* comprised the minority phyla (< 2.5% each). At the genus level, *Streptococcus* was the most abundant genus (19.0% abundance; phylum *Firmicutes*), followed by *Fusobacterium* (10.9% abundance; phylum *Fusobacteria*), *Actinomyces* (9.7% abundance; phylum *Actinobacteria*), *Prevotella* (8.5% abundance; phylum *Bacteroidetes*), and *Veillonella* (7.1% abundance; phylum *Firmicutes*). Dataset was rarefied to 8,000 reads per sample, and samples with shallow sequencing depth (12 samples from 11 different sites, or 4.8% of all samples) were excluded from subsequent analyses.

**Figure 1:**
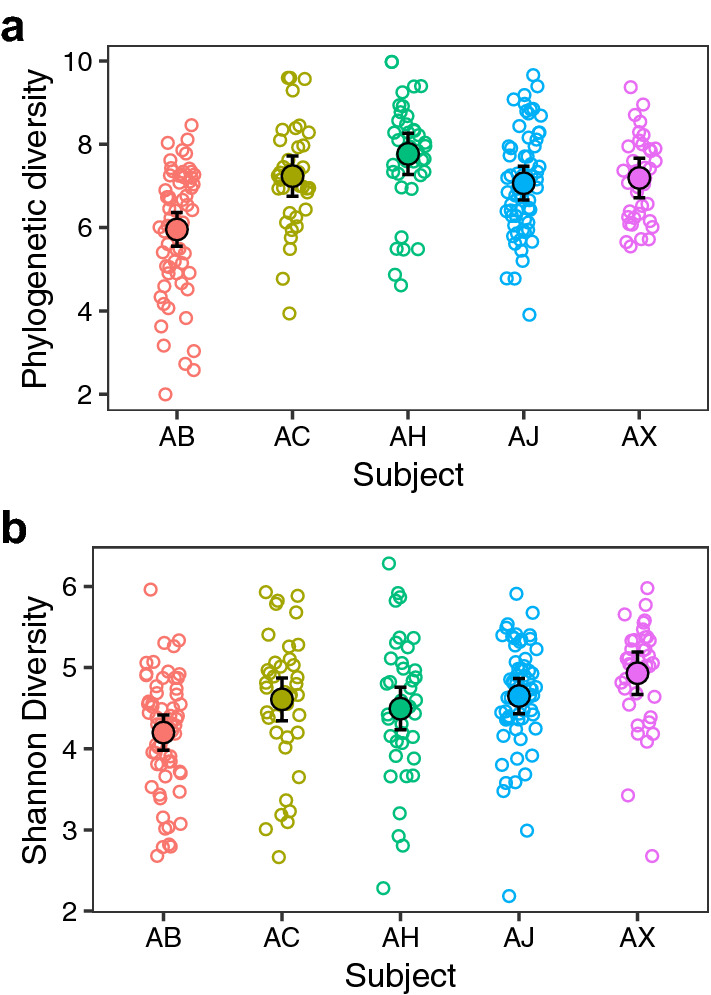
Alpha diversity of full-mouth subgingival microbiome in five periodontally healthy individuals. The alpha diversity of all subgingival samples is shown and the mean is indicated by the large, solid circles. Vertical lines represent 95% confidence intervals. Given the non-independence of samples from the same sites or the same time points, mixed linear models were used to compare species richness and diversity across subjects.

We first calculated species richness (Faith’s phylogenetic diversity) and diversity (Shannon diversity) for individual samples in the overall dataset. The average richness and diversity was 7.04 and 4.58, respectively (Fig. 1). Both species richness and diversity differed among subjects. Species richness was significantly lower in Subject AB compared to the other four subjects (p < 0.01, Supplementary Table S1). Shannon diversity was also lower in Subject AB compared to the others (p < 0.05), except for Subject AH (Supplementary Table S1). Within each subject, the variability in microbiome richness was high between different subgingival sites (sd = 0.547), compared to the relatively lower variability in samples collected over time (sd = 0.078). Similarly, for species diversity, temporal variability of a given site (sd = 0.098) was lower than the variation between different sites (sd = 0.157). Taken together, these results demonstrate that species richness and diversity of subgingival microbiome differ among periodontally healthy individuals. Furthermore, temporal variation is low compared to spatial variation (i.e. different subgingival sites), indicating that subgingival microbiome in periodontally healthy individuals is stable over time.

### Subgingival microbial community membership and structure

**Figure 2:**
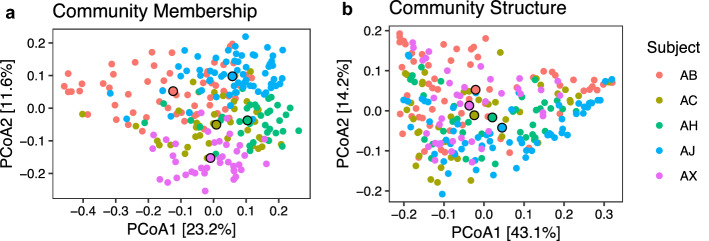
Community membership and structure of subgingival microbiome across subgingival pockets in five periodontally healthy individuals. Principal Coordinate Analysis of (**a**) unweighted UniFrac and (**b**) weighted UniFrac distances of subgingival samples from five subjects. Each point represents a single subgingival microbiome sample. For each subject, the centroid of all their samples is indicated by a larger outlined point.

We examined inter-individual and intra-individual (i.e. between different subgingival sites within each individual) differences in microbial community membership and structure, using unweighted and weighted UniFrac distance metrics. Unweighted UniFrac distances describe differences in community membership by comparing the proportion of shared phylotypes between samples. Weighted UniFrac distances describe differences in the community structure by accounting for the relative abundance in addition to the proportion of shared phylotypes. PERMANOVA of weighted and unweighted UniFrac distances showed that both community structure and membership of subgingival microbiome differed significantly between individuals (Table 1, Fig. 2), with subject identity explaining greater variation in the subgingival community membership compared to structure (Adonis; p < 0.01, R^2^ = 0.234 for community membership; R^2^ = p < 0.01, 0.168 for community structure). Indeed, clustering according to subject identity was more evident in the unweighted UniFrac analysis (Fig. 2a) compared to the weighted UniFrac (Fig. 2b), suggesting that minority OTUs differentiate individuals. The subgingival microbiome of individuals separated primarily along the second PCoA axis, although some overlap was observed between subjects. Within each individual, microbiomes from different subgingival sites differed significantly in both community membership (Adonis; p < 0.01, R^2^ = 0.295; Table 1, Supplementary Fig. 1) and structure (Adonis; p < 0.01, R^2^ = 0.347; Table 1, Supplementary Fig. 2). Site identity explained greater variation in community structure compared to membership, suggesting that site-to-site variation was driven primarily by the dominant members of the microbiome. The time of sampling contributed to only a small amount of variation to community membership and structure (Table 1). Analysis of the longitudinal dataset showed that the differences observed between subjects and between sites were also stable over time.

**Table 1:** Predictors of variation in microbiome community structure and membership using PERMANOVA.

	Weighted UniFrac (structure)	Unweighted UniFrac (membership)
Subject	R^2^ = 0.153, p = 0.001	R^2^ = 0.230, p = 0.001
Site in subject	R^2^ = 0.347, p = 0.001	R^2^ = 0.295, p = 0.001
Month in subject	R^2^ = 0.026, p = 0.038	R^2^ = 0.042, p = 0.001

Weighted UniFrac distances were used as a measure of differences in community structure while unweighted UniFrac distances were used as a measure of differences in community membership.

### Temporal stability of the subgingival microbiome

**Figure 3:**
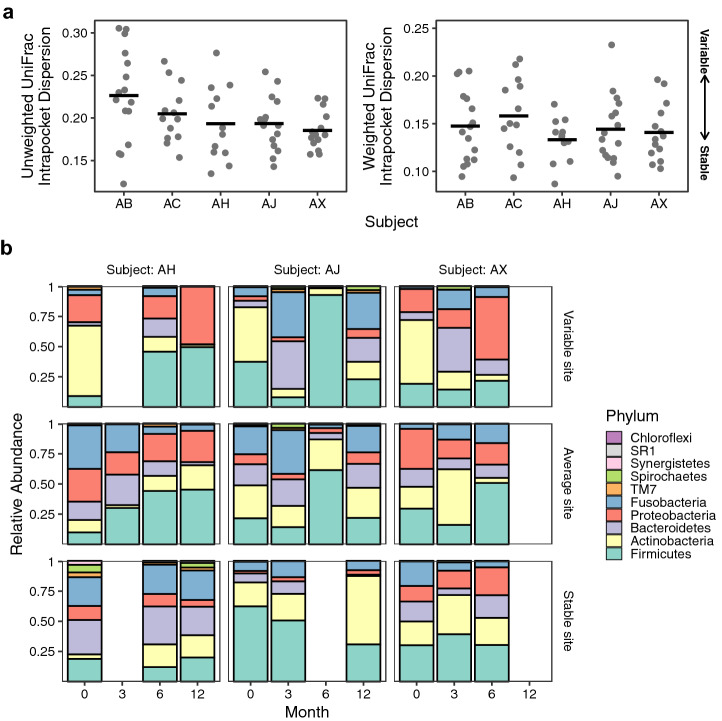
Temporal stability of subgingival microbiome. (**a**) Temporal stability was assessed as the mean UniFrac distance from each sample to the centroid of all samples from a given site. Each microbiome sample is a point, and black bars represents the mean for each subject. Small intra-site distances indicate more stable microbial communities over time, whereas large distances indicate more dynamic communities. (**b**) Relative abundance of subgingival microbiome at the phylum level is shown over time from baseline to the fourth visit (Visit 0: baseline; Visit 1: 3 months; Visit 2: 6 months; Visit 3: 12 months). Data from three representative subjects (AH, AJ, and AX) are shown to demonstrate different levels of microbiome stability at different subgingival sites. Each panel corresponds to one of the colored data points outlined with a black circle in panel (**a**), and represents the longitudinal microbiome data from a single subgingival site. Data for all sites are shown in Supplementary Fig. 3.

To examine temporal stability of the subgingival microbiome, we measured the dispersion of unweighted and weighted UniFrac distances for each subgingival site sampled longitudinally. Low UniFrac dispersion among samples collected over times suggests temporal stability, whereas higher dispersion indicates more variability or decreased stability. One site from subject AX was excluded from the analysis since only one time point was available. We observed considerable variations in the temporal stability of community membership and structure among different subgingival sites (Fig. 3a). However, the mean dispersion did not differ significantly between individuals for either weighted or unweighted UniFrac metric (ANOVA, p > 0.1; Fig. 3a). Compared to more variable sites that are characterized by large shifts in microbial composition at the phylum level, changes in stable sites were minimal (Fig. 3b, Supplementary Fig. 3). Interestingly, both stable and variable sites were observed in all subjects (Fig. 3, Supplementary Fig. 3).

**Figure 4:**
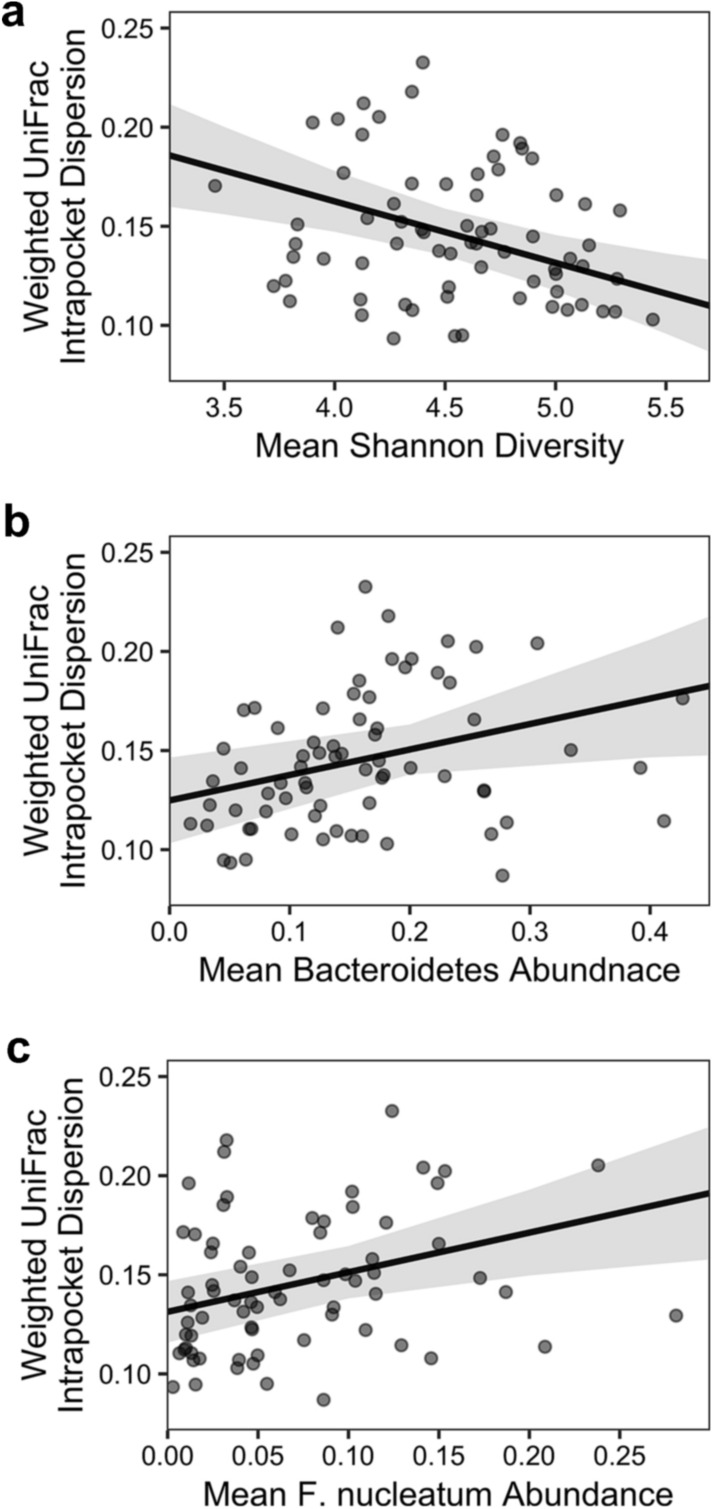
Factors associated with temporal stability of subgingival microbiome. Each point represents a single subgingival site. Temporal stability was assessed using mean UniFrac distance from each sample to the centroid of all longitudinal samples collected from the same site. Shannon diversity, *Bacteroidetes* abundance, and *F. nucleatum* abundance for a given site were determined by averaging data from all samples collected from the same site. The black line shows the predicted linear mixed model, and the shaded area represents the 95% confidence interval. Model coefficients are listed in Table 1.

To identify microbiome factors associated with stability of subgingival community structure, we examined the association between Shannon diversity, *Bacteroidetes* abundance, or *F. nucleatum* abundance with microbiome stability. Using linear mixed models, sites with higher Shannon diversity (b = − 0.03 ± 0.009SE, p < 0.01; Fig. 4a, Table 2) and lower abundance of *Bacteroidetes* (b = 0.12 ± 0.057SE, p = 0.032; Fig. 4b, Table 2) were significantly associated with temporal stability. The abundance of *Fusobacterium nucleatum* was not a significant predictor (p = 0.44; Table 2). However, when *Bacteroidetes* was removed from the model, increased *F. nucleatum* abundance was significantly associated with community instability (b = 0.20 ± 0.068SE, p < 0.01; Fig. 4c, Table 2). Thus, higher microbial diversity correlated positively with temporal stability, but the abundance of *Bacteroidetes* and *Fusobacterium nucleatum* correlated negatively with stability.

**Table 2:** Mixed linear models testing the association between community structure stability and microbiome predictors.

	Complete model	Reduced model
Shannon Diversity	b = − 0.031 ± 0.009SE, p < 0.01	b = − 0.024 ± 0.009SE, p < 0.01
*F. nucleatum*	b = 0.07 ± 0.089SE. p = 0.44	b = 0.20 ± 0.068SE, p < 0.01
*Bacteroidetes*	b = 0.12 ± 0.057SE, p = 0.032	

*F. nucleatum* and *Bacteroidetes* mean abundances over all sampling periods were used as predictors. The “complete model” includes all predictors, while the “reduced model” removes *Bacteroidetes* mean abundance.

### Stability of subgingival metagenome

**Figure 5:**
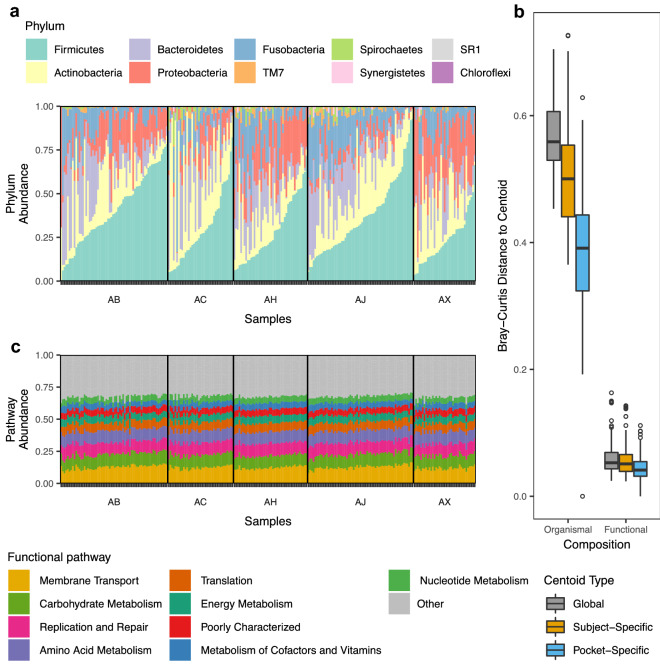
Inter- and intra-individual variability of subgingival microbiome composition and the predicted gene functions. (**a**) Relative abundance of microbiome at the phylum level across samples. (**b**) Relative abundance of the predicted gene functions across samples. (**c**) Bray–Curtis distances for “Organismal” (microbial composition) or “Functional” (metagenomic potential) were calculated between all subgingival samples and the centroids indicated (Global, Subject-Specific, or Pocket-Specific). “Global” distances indicate the distances from samples to the centroid of all samples. “Subject-Specific” distances are the distances between all samples from the same subject and the centroid for that subject. “Pocket-Specific” distances indicate the distances between all temporal samples from the same subgingival pocket and the centroid of that pocket. The median and interquartile range (IQR) are shown as boxplots. Whiskers show 1.5 * IQR and hollow points are outliers.

Given the variations in microbiome composition between individuals and between sites within individuals (Fig. 3a), we asked whether the functional potential encoded by the microbiome also differed between sites and individuals. Using PICRUSt, a bioinformatics tool to predict gene functions based on 16S rRNA information, we examined inter- and intra-individual variations in the metabolic functions of the subgingival microbiome. The functional metagenome of 14 samples (11 from subject AM and 3 from subject AX) could not be predicted and thus were not included in the analysis. A total of 328 gene categories were identified, belonging to 41 functional groups. Overall, non-ABC transporters (6.0%), ABC transporters (3.4%), and DNA repair proteins (3.2%) were the most abundant gene categories. Of the larger functional groups, membrane transport was the most abundant (11.8%), followed by DNA replication and repair (9.7%), carbohydrate metabolism (9.5%), and amino acid metabolism (9.1%). Surprisingly, despite the large inter- and intra-individual variations in the microbial composition (Fig. 5a), the predicted metagenome was remarkably similar both between individuals and between sites within individuals (Fig. 5b).

To compare temporal dynamics of the microbiome and metagenome, we calculated Bray–Curtis distances using microbiome and metagenome datasets, and generated three different types of centroids to measure dispersion: distance from each sample to the centroid of all samples (global centroid), distance from each sample from a subject to the centroid of that subject (subject-specific centroid), and distances from samples collected longitudinally from the same subgingival site to the centroid of that site (pocket-specific centroid). This analysis revealed that the metagenomes of samples collected over time from the same subgingival site were most similar to one another, whereas metagenomes between different subjects were most different (Fig. 5c). In all comparisons, inter- and intra-individual differences in the predicted metagenome were considerably smaller compared to the differences in the microbiome (Fig. 5c), indicating a high degree of functional similarity in the metagenome despite the highly personalized microbiome composition.

## Discussion

Nearly half of adults over the age of 30 in the United States suffer from some forms of periodontal disease^25^. The development of periodontitis has been associated with dysbiosis of subgingival microbiome^7–9,22^. Many studies have examined the subgingival microbiome in periodontally healthy individuals as well as individuals with various stages of periodontal disease. However, most published studies have been cross-sectional^7–10,22^, and longitudinal studies of subgingival microbiome describing the natural variations and potential pathogenic fluctuations have been limited. Since subgingival sites are often sampled from a few selected sites or pooled from multiple sites to represent overall periodontal health, the degree to which subgingival microbiome varies from site to site within a given individual has not been well defined. We analyzed 251 subgingival samples from 5 periodontally healthy individuals over six or twelve months using 16S rRNA sequencing. Our analysis revealed that the healthy subgingival microbiome is highly personalized in microbial composition, but their encoded functions are remarkably similar. Within each periodontally healthy individual, subgingival microbiome differs significantly between sites. However, for each site, microbiome is relative stable over time. These results expand our current understanding of the spatial and temporal complexities of the subgingival microbiota, and provide guidance for sampling strategies in future oral microbiome studies.

Consistent with previous studies, we found that the healthy subgingival microbiome is a diverse community dominated by members of 5 major phyla^7,9,22^. The Shannon diversity of subjects in our study was higher than the previous estimates of healthy subgingival communities^7,8,22^. These differences may be a result of differences in sequencing technologies and/or sequencing depths. Several studies have shown that microbial communities of healthy sites are less diverse than those of diseased periodontal pockets^7,8,22^. Interestingly, the microbiome of subject AB, who had the healthiest clinical measurements, was considerably less diverse than the other four subjects. Given that these differences persisted over six months and all 5 subjects were periodontally healthy throughout, these differences in diversity may reflect other unknown environmental or host genetic factors.

Island biogeography theory has been useful in explaining spatial patterns of the human microbiome. Island biogeography theory posits that early colonizers can have profound effects on future community through Founder’s effect^1^. In this study, we observed that subject identity had a strong effect on subgingival microbial community, which was especially evident in community membership compared to community structure. These results indicate that our subjects shared the dominant organisms but hosted unique, minority OTUs that may have been acquired during early development^1^ or via maternal transmission^26^. Thus, the subgingival “microbial fingerprint” of an individual is characterized by the presence of minority OTUs rather than the shared, dominant organisms. Island biogeography theory also describes a distance effect where islands closer together are more similar due to higher rates of immigration and emigration. We found that subgingival sites within an individual differed in microbial composition, but intra-individual site-to-site variations were considerably less than inter-individual variations. In addition to the proximity between sites within the same individual, salvia acts as a bridge to increase bacterial exchange between different sites. Thus, the community composition likely depends more on environmental conditions of the different sites than a Founder’s event. Indeed, the pocket effect better explained community structure compared to community membership, indicating that differences between sites were driven by the abundance of dominant organisms rather than the presence of minority species. Interestingly, we note that Proctor et al.^27^ observed an ecological gradient of various tissue types in the oral cavity from the front to the back of the mouth and on the exposed tooth surfaces that are shaped by salivary flow. While the differences in microbial communities between molars and incisors, and between buccal and lingual samples explained some of the variations observed in the gradient, the interpersonal variations accounted for the majority of the total variance among their samples, consistent with the large inter-individual variations observed in our study.

Inter-individual differences in the microbiome have been widely observed across different body habitats^2,11–13^. Previous studies have identified microbial differences between different body sub-habitats, but these sub-habitats often have different physiological functions or environmental conditions^2,13,16,19^. For instance, Grice et al. demonstrate that the armpit (moist skin environment) and mid-forearm (dry skin environment) display distinct microbiomes due to different environmental conditions despite little separation in physical space^16^. Here, we showed that defined sites within the same habitat can also harbor unique communities, likely due to small differences in the local conditions. These local conditions could be attributed to minor differences in probing depth or clinical attachment loss. We could not test for associations between tooth type (i.e. molars, incisors) or tooth surface (i.e. buccal, lingual) that may explain the between-pocket differences, as more extensive sampling is necessary to determine whether between-pocket variations reflect a stochastic process or environmental differences attributable to clinical measures, tooth type, or tooth surface.

Longitudinal microbiome studies have become increasingly important in order to understand dysbiotic processes and examine causality. Over six to twelve months, we found that between-subject and between-site differences were relatively stable. While these differences persisted over time, we found that every subject has both stable and more variable sites, and different sites differed in their degree of temporal stability. We did not find that individuals differed in their mean level of stability, although this has been reported for other body habitats. Flores et al. extended properties of microbial community such as species turnover to the concept of personal microbiome after finding individuals varied in their level of stability over three months^15^. More intense sampling will be necessary to examine differences in stability between individuals. However, these differences could also reflect short-term stochasticity rather than a long-term characteristic of a person. For instance, the gut microbiome diverges from its original community over time, with the divergence eventually reaching a plateau^28^. Dense and long-term sampling will be necessary to determine if individuals have different plateaus or just different rates of reaching a similar plateau.

Despite similar levels of stability across individuals, we identified several factors that contributed to temporal stability. Increased Shannon diversity led to increased community stability within subjects. The diversity-stability relationship has been examined in ecological systems both at the macro and micro scale^29^, and recent microbiome studies have shown similar results^13–15^. More diverse communities are likely to saturate available niches, which stabilize the community by minimizing opportunities for new and/or potentially pathogenic colonizers to establish. Furthermore, sites with higher *Bacteroidetes* and *Fusobacterium nucleatum* abundance were less stable. Enrichment of *Bacteroidetes* in diseased sites, compared to healthy sites, has been shown in some studies^7,9^. *Bacteroidetes* contains several members of the red and orange complex, which have been strongly associated with periodontal disease^23^. A member of the orange complex, *Fusobacterium nucleatum,* belongs to the phylum *Fusobacteria* and is known as the “bridge species,” assisting the succession of a healthy subgingival community to a pathogenic state^30^. *Fusobacterium nucleatum* is also associated with other dysbioses throughout the gastrointestinal tract, raising the possibility that its destabilizing properties are not limited to the oral cavity^24^. The higher abundance of pathogenic organisms and community instability may reflect an early transition from a healthy commensal-rich community toward a more pathogenic, dysbiotic state.

A paradigm shift in microbiome research is emerging, moving away from identifying members of the microbiome and classifying taxonomy towards understanding gene functions conferred by the community. The predicted metagenome of healthy subgingival community is dominated by membrane transport, DNA synthesis, and carbohydrate metabolism. Even though PICRUSt analysis only estimates metagenomic functional potential, these findings support the previous work^10^ using whole genome shotgun sequencing which showed that a majority of genes in the subgingival microbiome are involved in carbohydrate metabolism or protein, RNA, and DNA biosynthesis. We observed that the predicted metagenome was considerably more stable across individuals and time compared to the microbiome. Similar results have been shown in other body habitats^1,3^. In the gut, core members of the microbiome are few, but many gene functions are shared across individuals composing a core metagenome^3^. This high degree of similarity in the subgingival metagenome suggests that gene functions are redundant across different bacteria, which could replace each other with minor functional consequences^10^. As PICRUSt only predicts gene functions, our results will need to be confirmed by metagenomic sequencing in future studies. Furthermore, comparative studies between health and disease are warranted to further assess the importance of temporal stability of metagenome.

This study has limitations. Since our study focused on intense sampling and single-site resolution in the whole mouth over several time points within a 1-year follow up, the small number of subjects (n = 5) in our study precluded the ability to adequately evaluate chronological age or gender as variables on microbiome variation. Future work should include efforts to sample a larger cohort with a wide distribution of chronological age and an equal number of males and females to quantify the effect of each on the structure of subgingival microbial communities.

In summary, we surveyed the subgingival microbiome of five periodontally healthy individuals extensively. Each individual harbored a unique microbiome but shared typical subgingival microorganisms. Subgingival communities from different sites within an individual harbored different communities, and these differences were generally stable over time. We observed a high degree of temporal stability at each subgingival site, but sites with lower diversity and more abundant pathogenic organisms were less stable over time. Despite the clear differences in microbiome composition between individuals, the predicted subgingival metagenome was remarkably similar across individuals, sites, and time. These results highlight the ecological complexities that govern the spatial and temporal dynamics of the subgingival microbiome and suggest that a single measurement of the subgingival microbiome at a given site can provide long term information of the microbial composition and functional potential. However, sampling of each subgingival site is necessary to determine the microbiome composition of individual sites.

## Methods

### Subject recruitment and sample collection

Participants were recruited from the Periodontology Clinic and the DMD Student Dental Clinic at University of Florida College of Dentistry, Gainesville, FL., from March 2012 to March 2013. Subjects were included in the study if they met the following criteria: age > 18, a minimum of 20 natural teeth, and a healthy periodontal status at each visit. Periodontal health was determined using the CDC-AAP case definition^31^ with one modification. A patient was considered periodontally healthy if all pockets sampled had a probing depth ≤ 4 mm or a clinical attachment loss ≤ 3 mm, even if one site probed ≥ 5 mm. Other exclusion criteria included: tobacco smoking, diabetes, pregnancy, lactation, systemic antibiotic use within 6 months prior to enrollment, periodontal treatment within the previous 12 months, known immunodeficiency, or use of any immunosuppressive medications.

For each subject, clinical measurements (i.e. pocket depth, clinical attachment loss, plaque index) were collected and at least 12 subgingival sites were sampled at the initial visit, and again at three and six months. Three subjects also had samples collected at 12 months (Supplementary Table S2). Biofilms on the supragingival surface were removed using sterile gauze prior to subgingival sample collection. Samples from subgingival sites were collected using sterile endodontic paper points, and transferred to sterile tubes containing sample buffer (MO BIO, Carlsbad, CA), placed immediately on ice, and stored at − 80 °C until DNA extraction.

Informed consent was obtained from all patients for study participation and procedures. The study was approved by the Institutional Review Board at the University of Florida under project #444-2011. All experiments were performed in accordance with relevant guidelines and regulations.

### DNA extraction, PCR amplification, and Illumina sequencing

Genomic DNA was extracted using the Mobio (Carlsbad, CA) PowerSoil DNA extraction kit according to the manufacturer’s instructions. For each sample, the V1-V3 hypervariable region of the 16S rRNA gene was amplified using composite 27F (5′ AGAGTTTGATCCTGGCTCAG 3′) and 534R (5′ ATTACCGCGGCTGCTGG 3′) primers. Each 20 µL PCR reaction mixture contained 4 µL of extracted DNA, 100 nM of the forward primer, 100 nM of the reverse primer, and 10 µL of SuperFi PCR master mix (Invitrogen, Carlsbad, CA, USA). PCR products were analyzed on 1% SYBR Safe agarose gel. Gel slices containing the amplicons were extracted and purified using the Qiagen gel extraction kit (Qiagen, Valencia, CA, USA). Purified PCR products were quantified using the Qubit HS DNA quantification kit (Invitrogen, Carlsbad, CA, USA) and pooled in equimolar concentration. qPCR was used to quantify the concentration of the pooled PCR products and prepare the library for sequencing. The use of barcodes allowed multiplexing and bidirectional sequencing on the Illumina MiSeq platform (Illumina, San Diego, CA, USA).

### Data processing

Raw MiSeq paired-end reads of 300 nt each (covering the V1–V3 hypervariable region of the 16S rRNA gene using primers 27F and 534R) were processed using custom scripts written in R^32^. The reads were filtered based on exact matches to barcode/primer and an average quality score of 30. Samples were de-multiplexed according to the combination of their unique variable length barcodes (4 to 8 nt) on each paired end. Any OTUs that did not have at least one sample meeting a threshold abundance of 0.05% was excluded. In downstream analysis, the barcodes and primers (27F and 534R) were trimmed. To reconstruct the original contiguous amplicon, paired end reads were joined using FLASh, with a minimum overlap of 10 bp. USEARCH alignment was employed with a 97% identity and 80% aligned query threshold to assign reference OTUs (operational taxonomic units) with taxonomic information from the Human Oral Microbiome Database v.10.1^33^ to each joined read. Reads that did not meet filtering criteria were excluded from downstream analysis.

OTU tables from sequencing runs were merged. Singletons were filtered out, and samples with fewer than 20 total reads were excluded. Relative abundance was calculated from the unrarefied OTU table. The OTU table was then subsampled down to an even sequencing depth of 8,000 reads. Alpha diversity and beta diversity metrics were estimated in QIIME2 (version 2018.8, https://qiime2.org/) using the core-metrics-phylogenetic pipeline. Alpha diversity was measured with species richness (Faith’s phylogenetic diversity) and species diversity (Shannon diversity). Differences between communities were measured using weighted and unweighted UniFrac distances. Temporal stability of microbial communities was estimated with the mean intra-pocket dispersion calculated using the betadisper() function in vegan v.2.5-3^34^, grouping by individual periodontal sites. Sites with greater mean intra-pocket dispersion indicate more variability over time whereas sites with lower dispersion were more stable.

Functional metagenome was predicted from the unrarefied OTU table using the PICRUSt (Phylogenetic Investigation of Communities by Reconstruction of Unobserved States)^35^ tool in the bioBakery Python package. Relative abundance of different gene functions and functional pathways was calculated. The Bray–Curtis distance metric calculated in QIIME2 was used to compare temporal and spatial patterns of the microbiome and their predicted metagenome after subsampling to 8000 reads and 12,000 genes per sample, respectively. Dispersion was estimated with betadisper() using the centroid of all samples, subject-specific centroids, and site-specific centroids.

### Statistical analyses

Statistical analyses were performed in R v.3.4.2^32^ unless otherwise noted. Differences in alpha diversity were analyzed with linear mixed models using the lmer() function in lme4 v.1.1-19^36^, and site identity and time point as random effects. Principal coordinates analysis (PCoA) of Unifrac distances were used to examine clustering of subjects. Statistical significance of clustering by subject or site identity was determined with Permutational Multivariate Analysis of Variance (PERMANOVA) using the Adonis() function in vegan v.2.5-3^34^, where site was nested within subject. Temporal stability was compared across individuals using an analysis of variance (ANOVA). The association between temporal stability and several predictors (Shannon diversity, *Bacteroidetes*, *Fusobacterium nucleatum*) was tested using mixed linear models, with subject identity as a random effect.

### Ethics approval and consent to participate

Informed consent was obtained from all patients for study participation and procedures. The study was approved by the Institutional Review Board at the University of Florida under project #444-2011.

## Supplementary Information

Supplementary Information 1.

Supplementary Information 2.

Supplementary Information 3.

Supplementary Information 4.

Supplementary Information 5.

Supplementary Information 6.

Supplementary Information 7.

Supplementary Information 8.

## Data Availability

All sequence data are available in DANS under https://doi.org/10.17026/dans-xsq-egmz (https://doi.org/10.17026/dans-xsq-egmz). Analyses and scripts are presented as a supplementary file.

## Abbreviations

OTU: Operational taxonomic unit
PERMANOVA: Permutational multivariate analysis of variance
PCoA: Principal coordinates analysis

## References

[1] Turnbaugh PJ (2007). The human microbiome project. Nature.

[2] Consortium HMP (2012). Structure, function and diversity of the healthy human microbiome. Nature.

[3] Turnbaugh PJ (2009). A core gut microbiome in obese and lean twins. Nature.

[4] Chang HW (2018). Alteration of the cutaneous microbiome in psoriasis and potential role in Th17 polarization. Microbiome.

[5] Morgan XC (2012). Dysfunction of the intestinal microbiome in inflammatory bowel disease and treatment. Genome Biol..

[6] Erb-Downward JR (2011). Analysis of the lung microbiome in the “healthy” smoker and in COPD. PLoS One.

[7] Griffen AL (2012). Distinct and complex bacterial profiles in human periodontitis and health revealed by 16S pyrosequencing. ISME J..

[8] Abusleme L (2013). The subgingival microbiome in health and periodontitis and its relationship with community biomass and inflammation. ISME J..

[9] Kirst ME (2015). Dysbiosis and alterations in predicted functions of the subgingival microbiome in chronic periodontitis. Appl. Environ. Microbiol..

[10] Dabdoub SM, Ganesan SM, Kumar PS (2016). Comparative metagenomics reveals taxonomically idiosyncratic yet functionally congruent communities in periodontitis. Sci. Rep..

[11] Caporaso JG (2011). Moving pictures of the human microbiome. Genome Biol..

[12] Costello EK (2009). Bacterial community variation in human body habitats across space and time. Science.

[13] Zhou Y (2013). Biogeography of the ecosystems of the healthy human body. Genome Biol..

[14] Mehta RS (2018). Stability of the human faecal microbiome in a cohort of adult men. Nat. Microbiol..

[15] Flores GE (2014). Temporal variability is a personalized feature of the human microbiome. Genome Biol..

[16] Grice EA (2009). Topographical and temporal diversity of the human skin microbiome. Science.

[17] Gajer P (2012). Temporal dynamics of the human vaginal microbiota. Sci. Transl. Med..

[18] Halfvarson J (2017). Dynamics of the human gut microbiome in inflammatory bowel disease. Nat. Microbiol..

[19] Hall MW (2017). Inter-personal diversity and temporal dynamics of dental, tongue, and salivary microbiota in the healthy oral cavity. NPJ Biofilms Microbiomes.

[20] Belstrøm D (2016). Temporal stability of the salivary microbiota in oral health. PLoS One.

[21] Vogtmann E (2018). Temporal variability of oral microbiota over 10 months and the implications for future epidemiologic studies. Cancer Epidemiol. Biomark. Prev..

[22] Liu B (2012). Deep sequencing of the oral microbiome reveals signatures of periodontal disease. PLoS One.

[23] Socransky SS, Haffajee AD, Cugini MA, Smith C, Kent RL (1998). Microbial complexes in subgingival plaque. J. Clin. Periodontol..

[24] Han YW (2015). Fusobacterium nucleatum: A commensal-turned pathogen. Curr. Opin. Microbiol..

[25] Eke PI, Dye BA, Wei L, Thornton-Evans GO, Genco RJ (2012). Prevalence of periodontitis in adults in the united states: 2009 and 2010. J. Dent. Res..

[26] Funkhouser LJ, Bordenstein SR (2013). Mom knows best: the universality of maternal microbial transmission. PLoS Biol..

[27] Proctor DM (2018). A spatial gradient of bacterial diversity in the human oral cavity shaped by salivary flow. Nat. Commun..

[28] Faith JJ (2013). The long-term stability of the human gut microbiota. Science.

[29] McCann KS (2000). The diversity–stability debate. Nature.

[30] Kolenbrander PE, London J (1993). Adhere today, here tomorrow: Oral bacterial adherence. J. Bacteriol..

[31] Eke PI, Page RC, Wei L, Thornton-Evans G, Genco RJ (2012). Update of the case definitions for population-based surveillance of periodontitis. J. Periodontol..

[32] Core R Team. A Language and Environment for Statistical Computing (2019). *R Foundation for Statistical Computing* vol. 2 https://www.R--project.org. Accessed 1 Mar 2019.

[33] Chen T (2010). The Human Oral Microbiome Database: A web accessible resource for investigating oral microbe taxonomic and genomic information. Database J. Biol. Databases Curation.

[34] Oksanen, J., Blanchet, F. G., Friendly, M., Kindt, R., Legendre, P., McGlin, D., *et al*. vegan: Community Ecology Package. (2018).

[35] Langille MGI (2013). Predictive functional profiling of microbial communities using 16S rRNA marker gene sequences. Nat. Biotechnol..

[36] Bates D, Mächler M, Bolker BM, Walker SC (2015). Fitting linear mixed-effects models using lme4. J. Stat. Softw..

