# Supplementary material for: Stability of healthy subgingival microbiome across space and time
Source: Sci Rep. 2021 Dec 14;11:23987. doi: 10.1038/s41598-021-03479-2 (PMC8671439; doi:10.1038/s41598-021-03479-2)
Supplement: Supplementary file 1 — Supplementary Information 1. [file 41598_2021_3479_MOESM1_ESM.docx]

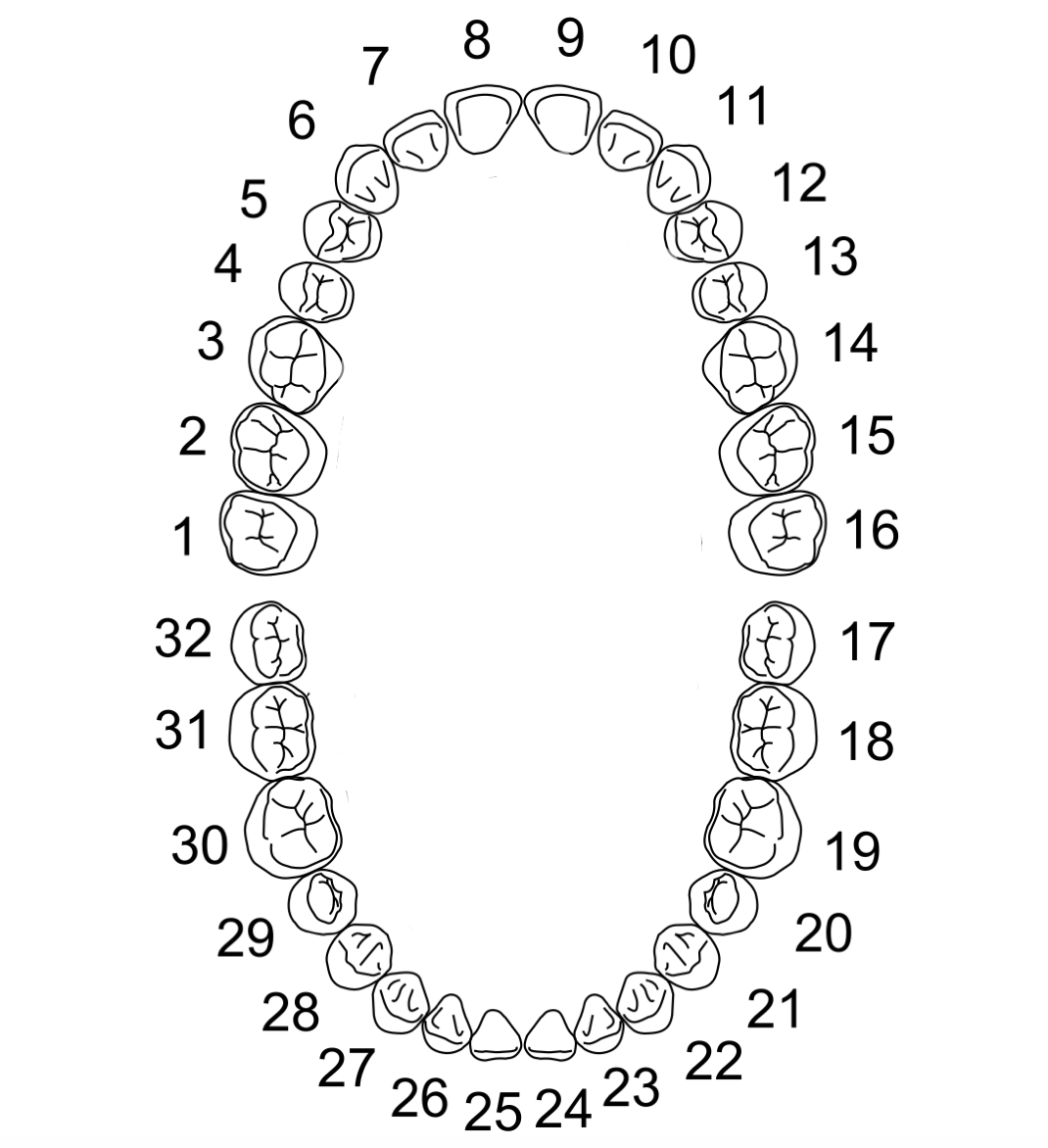


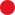

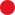

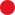

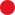

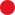

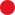

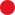

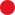

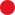

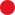

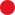

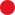

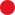

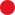

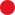

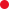

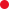

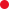

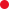

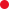

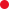

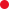

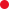

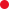

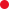

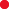

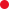

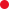

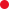

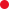

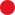

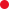


Subject AB


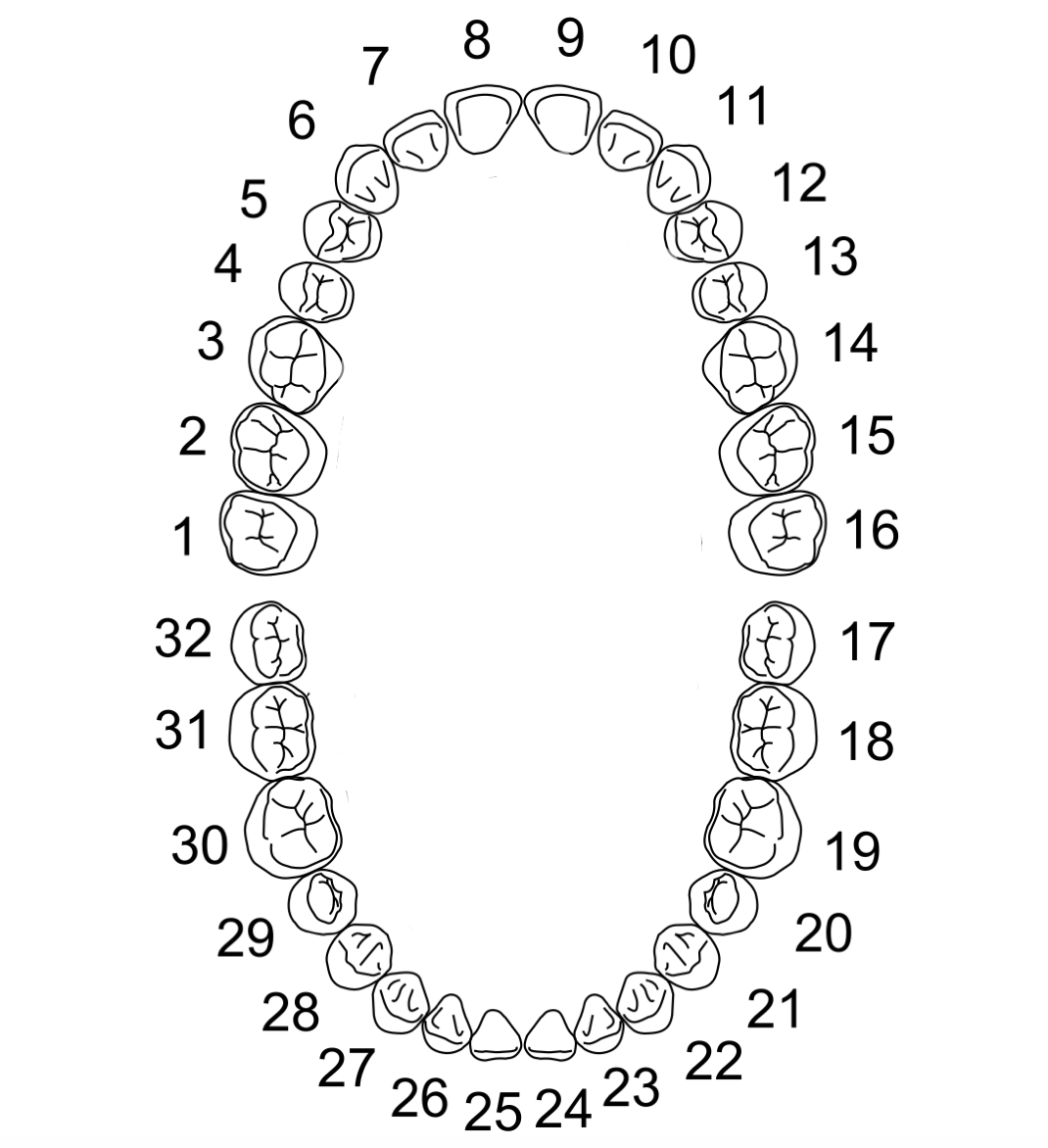


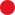

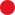

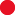

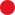

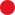

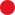

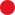

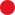

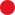

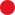

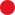

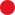

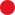


Subject AC


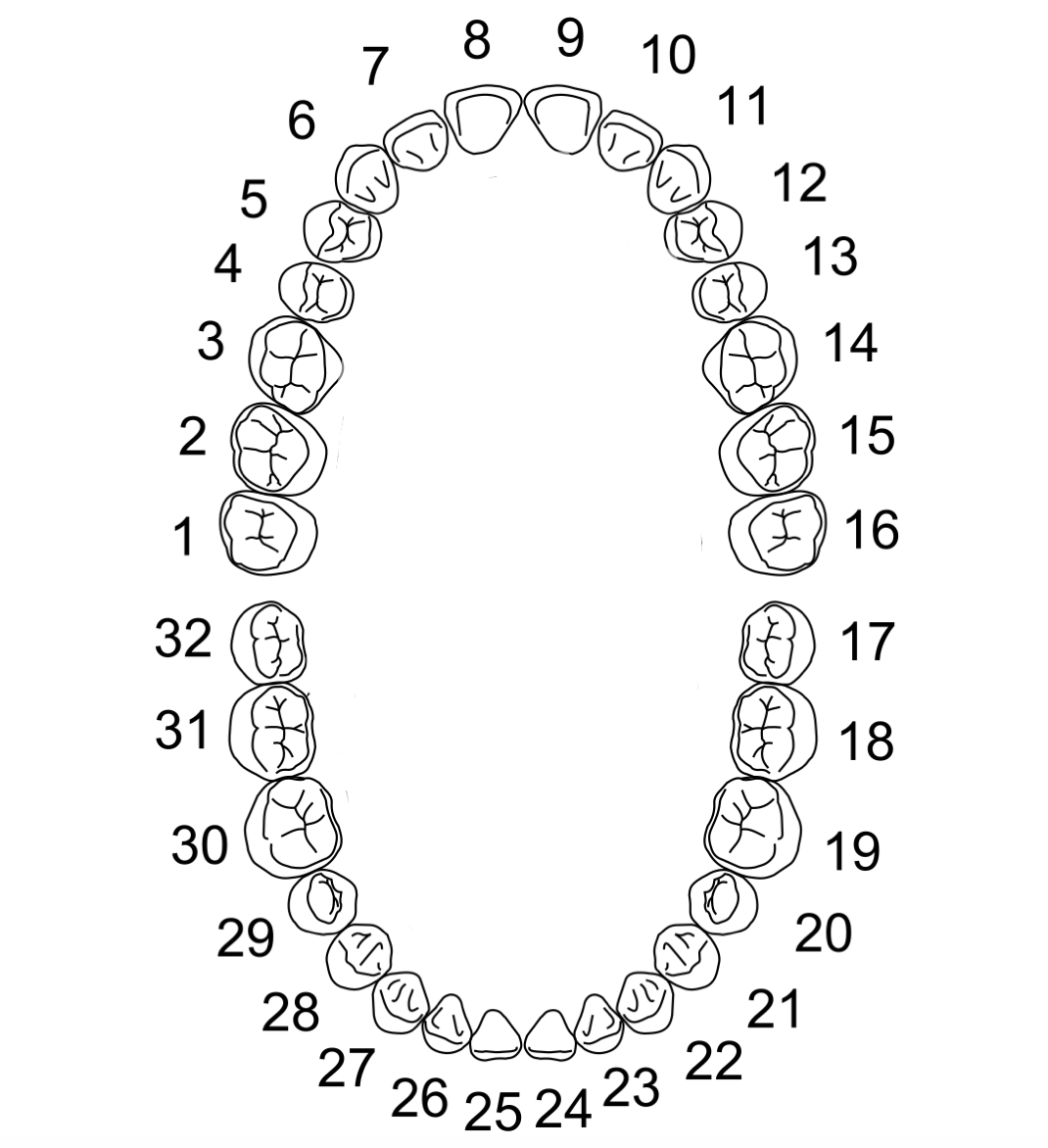


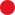

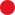

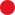

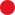

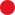

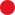

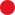

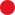

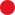

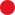

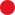


Subject AH


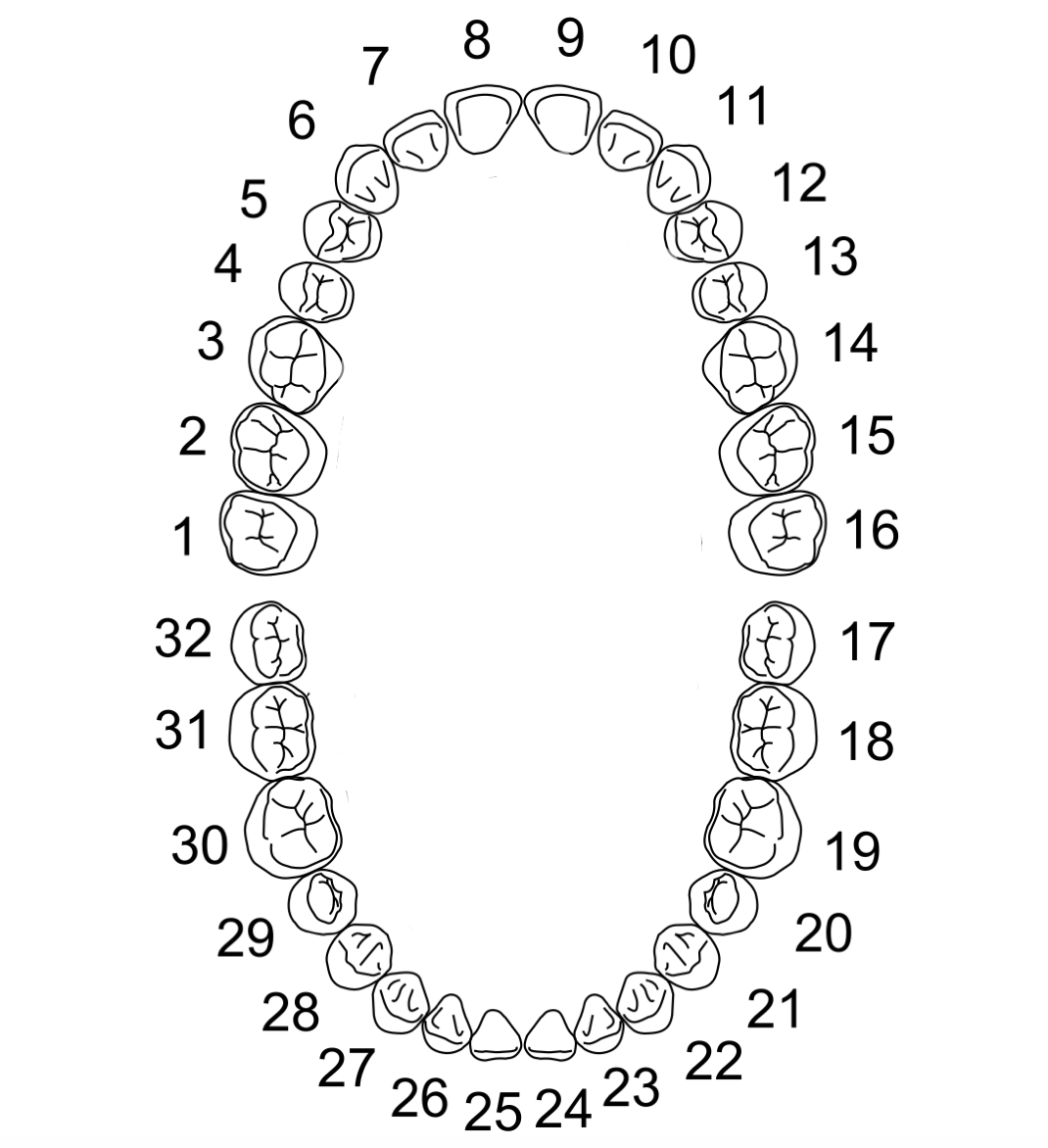


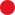

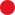

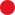

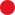

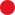

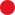

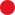

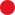

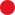

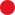

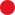

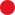

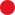

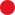

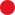


Subject AJ


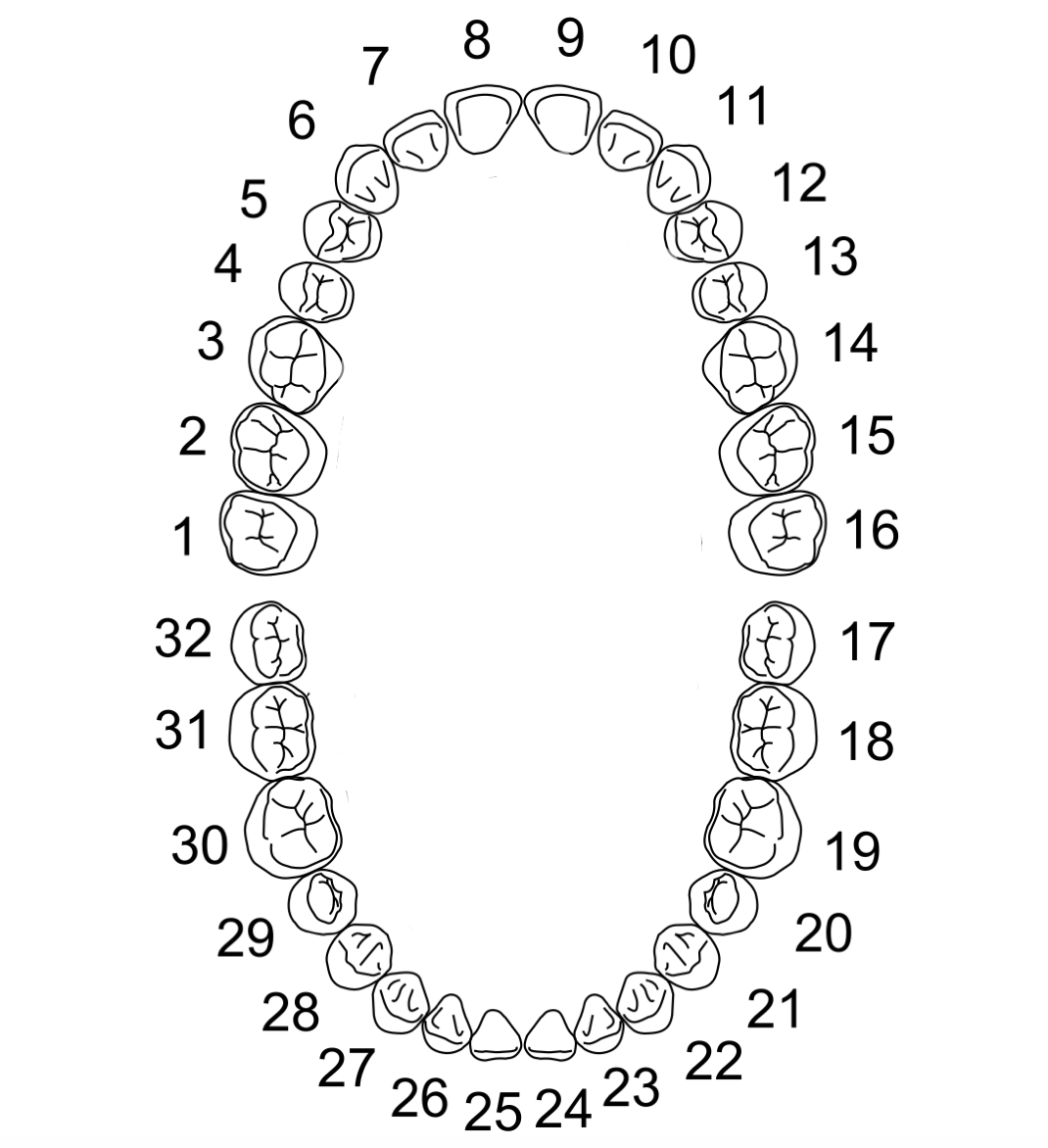


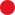

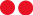

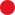

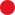

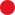

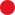

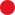

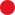

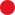

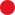

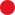

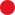


Subject AX

Supplementary Figure 4: Map of subgingival sampling sites for individual subjects. Each dot represents the relative location of repeated clinical measurements and microbial samples. Sites that have been collected for each subject are indicated in the Figure, which was adapted using the Universal dental numbering System file licensed under the [Creative Commons](https://en.wikipedia.org/wiki/en:Creative_Commons) [Attribution-Share Alike 3.0 Unported](https://creativecommons.org/licenses/by-sa/3.0/deed.en) license and is available at <https://commons.m.wikimedia.org/wiki/File:Universal_Numbering_System.svg#mw-jump-to-license>.
