# Supplementary material for: Stability of healthy subgingival microbiome across space and time
Source: Sci Rep. 2021 Dec 14;11:23987. doi: 10.1038/s41598-021-03479-2 (PMC8671439; doi:10.1038/s41598-021-03479-2)
Supplement: Supplementary file 7 — Supplementary Information 7. [file 41598_2021_3479_MOESM7_ESM.docx]

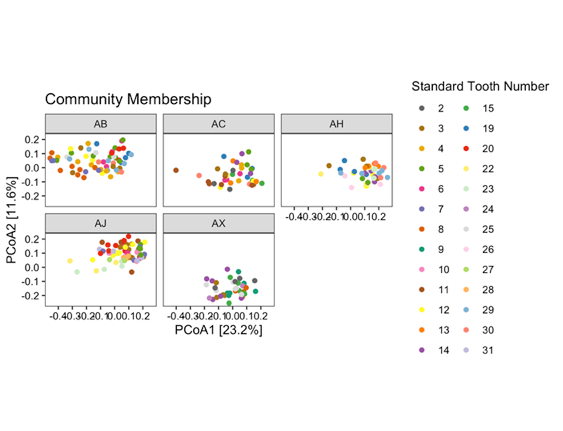


*Supplementary Figure 1.* **Community membership of subgingival microbiome across different subgingival sites in five periodontally healthy individuals.** Principal Coordinate Analysis of unweighted UniFrac distances of subgingival samples from five subjects. Samples were divided by subject, and each point represents a single subgingival sample. Samples collected over time from the same site are shown using the same color.


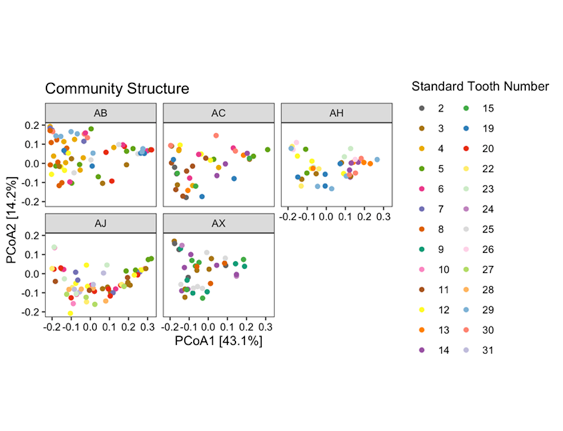


*Supplementary Figure 2.* **Community structure of the subgingival microbiome across subgingival pockets in five periodontally healthy individuals.** Principal Coordinate Analysis of weighted UniFrac distances of subgingival samples from five subjects. Each panel presents a subject. Each point represents a single subgingival sample. Samples collected over time from the same subgingival site are shown using the same color.

*Supplementary Figure 3.* **Temporal stability of subgingival microbiome varies from site to site within subjects.** Relative abundance of subgingival microbiome at the phylum level is shown on the y-axis. Each panel represents the longitudinal data of a single subgingival site, and is plotted over time from baseline to the fourth visit (left to right; visits 1 to 4). Sites (panel) are ordered from left to right according to relative stability within each subject (1-most stable, 16-most dynamic).
