# Supplementary material for: Stability of healthy subgingival microbiome across space and time
Source: Sci Rep. 2021 Dec 14;11:23987. doi: 10.1038/s41598-021-03479-2 (PMC8671439; doi:10.1038/s41598-021-03479-2)
Supplement: Supplementary file 8 — Supplementary Information 8. [file 41598_2021_3479_MOESM8_ESM.docx]

|  | Phylogenetic diversity | Shannon diversity |
| --- | --- | --- |
| Intercept (ref:AB) | b = 5.96 ± 0.21SE, p < 0.001 | b = 4.20 ± 0.11SE, p < 0.001 |
| AC | b = 1.28 ± 0.32SE, p < 0.001 | b = 0.41 ± 0.16SE, p = 0.012 |
| AH | b = 1.81 ± 0.32SE, p < 0.001 | b = 0.30 ± 0.16SE, p = 0.067 |
| AJ | b = 1.11 ± 0.29SE, p < 0.001 | b = 0.45 ± 0.14SE, p = 0.002 |
| AX | b = 1.23 ± 0.31SE, p < 0.001 | b = 0.73 ± 0.16SE, p < 0.001 |

*Table S1*. **Mixed linear models of alpha diversity metrics across subjects**. Subject AB was used as the reference level to which all other subjects were compared to. Model coefficients, standard errors (SE), and p values are given.

| Subject | AB | AC | AH | AJ | AX |
| --- | --- | --- | --- | --- | --- |
| Visits (months from baseline) | 4 (0/3/6/12) | 3 (0/3/6) | 4 (0/3/6/12) | 4 (0/3/6/12) | 3 (0/6/9) |
| Sites sampled per visit | 16 | 13 | 12 | 16 | 15 |
| Plaque index | 0.05 ± 0.28 | 0.28 ± 0.65 | 0.48 ± 0.63 | 0.94 ± 0.78 | 0.51 ± 0.78 |
| Probe depth (PD) | 2.63 ± 0.52 | 2.97 ± 0.36 | 3.09 ± 0.47 | 3.21 ± 0.57 | 2.71 ± 0.46 |
| Clinical attachment loss (CAL) | 0.00 ± 0.00 | 0.05 ± 0.22 | 0.05 ± 0.21 | 0.43 ± 0.56 | 1.78 ± 2.37 |
| Unique sites with PD > 3 | 1 | 2 | 4 | 9 | 0 |
| Unique sites with CAL > 2 | 0 | 0 | 0 | 0 | 15 |

*Table S2*. **Clinical Characteristics of Subjects**. For clinical measurements, mean ± standard deviation is shown.
